# Comparing species tree estimation with large anchored phylogenomic and small Sanger-sequenced molecular datasets: an empirical study on Malagasy pseudoxyrhophiine snakes

**DOI:** 10.1186/s12862-015-0503-1

**Published:** 2015-10-12

**Authors:** Sara Ruane, Christopher J. Raxworthy, Alan R. Lemmon, Emily Moriarty Lemmon, Frank T. Burbrink

**Affiliations:** Department of Herpetology, American Museum of Natural History, Central Park West at 79th Street, New York, NY 10024 USA; Department of Biology, Florida State University, 319 Stadium Drive, P.O. Box 3064295, Tallahassee, FL 32306-4295 USA; Biology Department, College of Staten Island/CUNY, 2800 Victory Boulevard, Staten Island, NY 10314 USA

**Keywords:** Madagascar, Anchored phylogenomics, Lamprophiidae, Next-generation sequencing

## Abstract

**Background:**

Using molecular data generated by high throughput next generation sequencing (NGS) platforms to infer phylogeny is becoming common as costs go down and the ability to capture loci from across the genome goes up. While there is a general consensus that greater numbers of independent loci should result in more robust phylogenetic estimates, few studies have compared phylogenies resulting from smaller datasets for commonly used genetic markers with the large datasets captured using NGS. Here, we determine how a 5-locus Sanger dataset compares with a 377-locus anchored genomics dataset for understanding the evolutionary history of the pseudoxyrhophiine snake radiation centered in Madagascar. The Pseudoxyrhophiinae comprise ~86 % of Madagascar’s serpent diversity, yet they are poorly known with respect to ecology, behavior, and systematics. Using the 377-locus NGS dataset and the summary statistics species-tree methods STAR and MP-EST, we estimated a well-supported species tree that provides new insights concerning intergeneric relationships for the pseudoxyrhophiines. We also compared how these and other methods performed with respect to estimating tree topology using datasets with varying numbers of loci.

**Methods:**

Using Sanger sequencing and an anchored phylogenomics approach, we sequenced datasets comprised of 5 and 377 loci, respectively, for 23 pseudoxyrhophiine taxa. For each dataset, we estimated phylogenies using both gene-tree (concatenation) and species-tree (STAR, MP-EST) approaches. We determined the similarity of resulting tree topologies from the different datasets using Robinson-Foulds distances. In addition, we examined how subsets of these data performed compared to the complete Sanger and anchored datasets for phylogenetic accuracy using the same tree inference methodologies, as well as the program *BEAST to determine if a full coalescent model for species tree estimation could generate robust results with fewer loci compared to the summary statistics species tree approaches. We also examined the individual gene trees in comparison to the 377-locus species tree using the program MetaTree.

**Results:**

Using the full anchored dataset under a variety of methods gave us the same, well-supported phylogeny for pseudoxyrhophiines. The African pseudoxyrhophiine Duberria is the sister taxon to the Malagasy pseudoxyrhophiines genera, providing evidence for a monophyletic radiation in Madagascar. In addition, within Madagascar, the two major clades inferred correspond largely to the aglyphous and opisthoglyphous genera, suggesting that feeding specializations associated with tooth venom delivery may have played a major role in the early diversification of this radiation. The comparison of tree topologies from the concatenated and species-tree methods using different datasets indicated the 5-locus dataset cannot beused to infer a correct phylogeny for the pseudoxyrhophiines under any method tested here and that summary statistics methods require 50 or more loci to consistently recover the species-tree inferred using the complete anchored dataset. However, as few as 15 loci may infer the correct topology when using the full coalescent species tree method *BEAST. MetaTree analyses of each gene tree from the Sanger and anchored datasets found that none of the individual gene trees matched the 377-locus species tree, and that no gene trees were identical with respect to topology.

**Conclusions:**

Our results suggest that ≥50 loci may be necessary to confidently infer phylogenies when using summaryspecies-tree methods, but that the coalescent-based method *BEAST consistently recovers the same topology using only 15 loci. These results reinforce that datasets with small numbers of markers may result in misleading topologies, and further, that the method of inference used to generate a phylogeny also has a major influence on the number of loci necessary to infer robust species trees.

**Electronic supplementary material:**

The online version of this article (doi:10.1186/s12862-015-0503-1) contains supplementary material, which is available to authorized users.

## Background

Phylogenetic studies are undergoing a massive jump in the scale of the molecular datasets used to estimate phylogenies, due to the ease of collecting hundreds of loci sampled throughout the genomes of non-model taxa (e.g., [1, 2]). Increasing the number of loci is expected to have a positive effect on phylogenetic estimation [3]. Both simulation and empirical studies [4–8] demonstrate strong correlation between the number of independent loci and phylogenetic accuracy, though the exact number of markers required to resolve relationships varies depending on the informativeness of the markers, the method of inference used, the number of taxa, and the time-scale being examined. Typically though, the ability to generate hundreds or thousands of loci that provide the appropriate amount of variation at differing time scales has been a challenge, especially across many non-model taxa. Recently, two different next-generation sequencing (NGS) protocols have produced large datasets composed of generally longer loci (in contrast to shorter length reads from restriction site-associated markers) useful for estimating phylogenies at varying temporal scales—the ultra-conserved element procedure of Faircloth et al. [9] and the anchored phylogenomics approach of Lemmon et al. [1]. These methods differ in the specific regions targeted and the numbers of loci produced, yet both produce orthologous markers across multiple, non-model taxa with substantial genetic variation for inferring phylogenies at both shallow and deep-time scales [1, 2, 7, 9–16].

While generating DNA datasets that cover the genome has become easier, estimating species-tree phylogenies with these data remains problematic [17]. Multi-species coalescent methods that jointly estimate gene trees and species trees, such as *BEAST [18], have proved robust for species-trees estimation. Although these full-coalescent methods can be highly accurate when using relatively few loci (e.g., [7]), they may not be suitable for the large numbers of loci produced using NGS techniques due to computational time and a lack of convergence as the number of taxa or loci increases [14, 19].

Alternatively, methods that use summarized information from user-provided gene trees to quickly estimate species trees, such as MP-EST [20] and STAR [21], are promising for analyzing NGS datasets. These methods can accommodate many taxa and loci and have the statistically desirable properties of being accurate when used with large numbers of loci and low levels of missing data [19, 20]. However, species-tree methods that depend on summarized gene-tree uncertainty may suffer when markers are short and uninformative [22, 23] or when incomplete-lineage sorting is not the main cause of gene-tree discordance [24–26]. While the concern of uninformative markers can be circumvented by using high quality datasets (i.e., longer loci with more informative sites), gene-tree discordance due to mutational variance or migration between species is a problem that still exists when estimating species trees even under a full-coalescent model such as *BEAST [24, 26, 27]. Despite these potential challenges, summary statistics approaches remain a viable option for NGS dataset analyses and several recent empirical studies with these types of data have used MP-EST and STAR to estimate well-resolved species trees that confirm previously hypothesized taxonomy as well as discover novel relationships (e.g., [13, 15, 28].

Here, we use an anchored phylogenomics dataset to construct a generic-level species tree for the Malagasy pseudoxyrhophiines and simultaneously explore how different datasets, with respect to locus number, influence phylogenetic inference. The subfamily Pseudoxyrhophiinae is part of the family Lamprophiidae, a mainly African radiation of snakes [29–31]. Pseudoxyrhophiines are among the most poorly studied of Colubroids, with little known with respect to ecology and reproduction (e.g., [32–34]), as well as basic morphology (e.g., hemipenial structure; [35]). This is unfortunate since pseudoxyrhophiines are unique among the world’s snake fauna as being the only island snake lineage where the majority of diversification takes place *in situ* on the island rather than through dispersal from the mainland [36], potentially making pseudoxyrhophiines an excellent model system for determining what factors promote ecological and morphological diversification within a closed system.

Of the currently recognized 89 species of pseudoxyrhophiine, 80 are endemic to Madagascar (excepting possible introductions to the Comoros; [37]), with the remaining taxa distributed in mainland Africa (five spp.), the Comoros islands (three spp.), and Socotra (one sp.). Previous studies have indicated the African and Socotran species are the sister lineage(s) to a monophyletic radiation of Malagasy/Comoros taxa [38, 39] but this has not been supported by the most recent phylogenetic estimates for the group [30, 31]. Prior molecular phylogenetic studies have included up to 54 of the recognized species of Pseudoxyrhophiinae for a single gene [40] and no study has used more than 10 loci to determine relationships among the genera, with the majority of taxa having only 1–6 loci available [30]. These studies also used concatenated gene-tree methodologies, rather than species-tree approaches, which are more likely to be misleading when using small numbers of loci [6]. Although under certain circumstances concatenation results in identical topologies when compared to a species trees [6, 41, 42], empirical studies have demonstrated that concatenation may overestimate branch lengths, causing inaccuracies in downstream phylogenetic analyses (e.g., [43]).

Using a NGS dataset comprised of 377 loci covering 77 % of the genera of Pseudoxyrhophiinae, we first estimate species trees using the full dataset and the summary statistics approaches STAR and MP-EST, and subsets of the loci using the multi-species coalescent method *BEAST. This is the first species tree to be produced for the subfamily, clarifying the intergeneric relationships of these snakes, and allowing us to more robustly examine the monophyly of the Malagasy genera, specifically with respect to the African mainland genus *Duberria*. Recent studies have found *Duberria* within a clade also containing the Malagasy pseudoxyrhophiine genus *Compsophis*, with this clade then sister taxon to the remaining Malagasy pseudoxyrhophiines [30, 31]. That relationship suggests that Malagasy genera are not a single monophyletic radiation, which is a key assumption for future studies examining diversification of these snakes in Madagascar [44]. Second, using varying numbers of loci with different tree-inference methodologies, we compare the tree topologies estimated from the full NGS dataset (377 loci) to smaller subsets of the NGS data (3–200 loci), as well as to a typical, Sanger-sequenced dataset comprised of five loci that are commonly used to infer squamate phylogenies. From these empirical results we determine if the numbers of loci and methods used influence phylogenetic inference for pseudoxyrhophiines.

## Methods

### Taxa sampled

We sampled 25 taxa: 23 pseudoxyrhophiines across 21 species, in 17 different genera and included two psammophiines as outgroups, *Mimophis mahfalensis*, and *Rhamphiophis rubropunctatus.* With the exception of the outgroup *Rhamphiophis rubropunctatus* and the pseudoxyrhophiine *Parastenophis betsileanus,* the same individuals were used for the Sanger and Anchored NGS methods (Additional file 1).

### Ethics statement

All sample collection complied with the policies and guidelines of relevant institutions, including the Ministries des Eaux et Forêts, Madagascar National Parks, the Université d’Antananarivo, Departement de Biologie Animale, the University of Michigan Museum of Zoology, and the American Museum of Natural History.

### DNA extraction and Sanger sequencing

DNA was extracted using a Quiagen® DNEasy kit, following the tissue protocol. Sanger sequencing was used to sequence five loci that have been previously utilized to infer squamate phylogenies [30, 43, 45]. This dataset consisted of two protein-coding mitochondrial genes (*COI*, 623 bp; *CytB*, 550 bp), two nuclear protein-coding genes (*Cmos* 562 bp; *Rag2*, 645 bp), and one nuclear intron (*Nav intron 5*, 610 bp) (PCR and sequencing details in Additional file 2). Sanger sequencing was performed at the American Museum of Natural History on an ABI 3730 sequencer. Sequences were edited and aligned using the Geneious alignment algorithm in Geneious® v.6.1.4 and checked by eye to ensure that protein coding loci did not contain stop codons.

### Anchored phylogenomics locus selection and probe design

We used the following approach to identify a set of loci that could be obtained efficiently in snakes using Anchored Hybrid Enrichment. First, in order to improve gene-tree resolution, we increased the size of each target region described in [1] to approximately 1350 bp by including flanking regions that contained sufficient sequence conservation between *Homo* and *Anolis*. Because some of the original anchor regions were near each other, some of these loci were joined to form a single locus. Loci that performed poorly in [1] were removed. The resulting locus set contained 394 loci comprising a total of 468,296 bp target region (referred to as the version 2 vertebrate loci; genomic coordinates available on Dryad doi:10.5061/dryad.kp400). In order to improve the capture efficiency for snakes, we also obtained homologous sequences from the *Python molurus* genome (NCBI accession AEQU000000000) and 15× genomic reads obtained for the brown reed snake *Calamaria pavimentata* (Illumina PE100bp; specimen obtained from California Academy of Sciences, accession CAS235364). After aligning the *Anolis*, *Python*, and *Calamaria* sequences using MAFFT [46], alignments were trimmed to produce the final probe region alignments (alignments available on Dryad doi:10.5061/dryad.kp400), and probes were tiled at approximately 1.5X tiling density per species (probe specification available on Dryad doi:10.5061/dryad.kp400).

The NGS dataset was generated by the Center for Anchored Phylogenetics [47] using the anchored hybrid enrichment methodology described by [1]. We used a Covaris E220 Focused-ultrasonicator to fragment each genomic DNA sample to a fragment size of ~150–350. A Beckman-Coulter Biomek FXp liquid-handling robot was then used to prepare indexed Illumina libraries following protocol modified from Meyer and Kircher [48] (with SPRIselect size-selection after blunt-end repair using a 0.9x ratio of bead to sample volume). A single pool containing all of the libraries was then enrichment for the target using an Agilent Custom SureSelect kit (Agilent Technologies) that contained the probes described above. The enriched library pool was then sequenced on 1 PE150 Illumina HiSeq2000 lane by the Translational Science Laboratory in the College of Medicine at Florida State University.

### NGS data assembly

Paired reads were merged following [49]. Briefly, for each degree of overlap for each read we computed the probability of obtaining the observed number of matches by chance, and selected degree of overlap that produced the lowest probability, with a *p*-value less than 10^−10^ required to merge reads. When reads were merged, mismatches are reconciled using base-specific quality scores, which were combined to form the new quality scores for the merged read (see [49] for details). Reads failing to meet the probability criterion were kept separate but still used in the assembly. Between 50 and 75 % of the sequenced library fragments had an insert size between 150 bp and 300 bp.

The reads for each sample were assembled into contigs using a reference assembly approach to map reads to the *Calamaria* probe regions and a de-novo assembly approach to extend the assembly into the flanks (java scripts available upon request from A. Lemmon). The reference assembler uses a library of spaced 20-mers derived from the sites conserved across squamates. A preliminary match resulted if at least 17 of 20 matches existed between the positions in a read and the corresponding positions in one of the spaced. Reads obtaining a preliminary match were then compared to the *Calamaria,* with greater than 55 matches out of 100 was considered a significant match. Approximate alignment positions of mapped reads were estimated using the position of the spaced 20-mers, and all 60-mers existing in the read were stored in a hash table used by the de-novo assembler. The de-novo assembler identifies exact matches between a read and one of the 60-mers found in the hash table. Simultaneously using the two levels of assembly described above, the read files were traversed repeatedly until an entire pass through the reads produced no additional mapped reads.

Mapped reads were then clustered for each locus into clusters using 60-mer pairs observed in the reads mapped to that locus. In short, a list of all 60mers found in the mapped reads was compiled, the 60-mers were clustered if found together in at least two reads. The 60-mer clusters were then used to separate the reads into clusters for contig estimation. Relative alignment positions of reads within each cluster were then refined in order to increase the agreement across the reads. Up to one gap was also inserted per read if needed to improve the alignment. Note that given sufficient coverage and an absence of contamination, each single-copy locus should produce a single assembly cluster. Low coverage (leading to a break in the assembly), contamination, and gene duplication, can all lead to an increased number of assembly clusters. A whole genome duplication, for example would increase the number of clusters to two per locus.

Consensus bases were called from assembly clusters as follows. For each site an unambiguous base was called if the bases present were identical or if the polymorphism of that site could be explained as sequencing error, assuming a binomial probability model with the probability of error equal to 0.1 and alpha equal to 0.05. If the polymorphism could not be explained as sequencing error, the ambiguous base was called what corresponded to all of the observed bases at that site (e.g. ‘R’ was used if ‘A’ and ‘G’ were observed). Called bases were soft-masked (made lowercase) for sites with coverage lower than 5. A summary of the assembly results is presented in the additional files (Additional file 3).

In order to filter out possible low-level contaminants, consensus sequences derived from very low coverage assembly clusters (<10 reads) were removed from further analysis. After filtering, consensus sequences were grouped by locus (across individuals) in order to produce sets of homologs. Orthology was then determined for each locus using a distance-based approach. First, a pairwise distance measure was computed for pairs of homologs by computing the percent of 20-mers observed in the two sequences that were found in both sequences. The list of 20-mers was constructed from consecutive 20-mers as well as spaced 20-mers (every third base) in order to allow increased levels of sequence divergence. Finally, we clustered the sequences using the distance matrix and a Neighbor-Joining algorithm that allowed at most one sequence per species to be assigned to a cluster. Only clusters containing at least 50 % of the species were utilized downstream.

Following orthology assessment, sequences in each orthologous set were aligned using MAFFT v7.023b [46]. The flags --genafpair and --maxiterate 1000 were utilized. The alignments for each locus was masked/trimmed in three steps. First, each alignment site was identified as “good” if the most common character observed was present in >50 % of the sequences. Second, 20 bp regions of each sequence that contained <10 good sites were masked. Third, sites with fewer than 4 unmasked bases were removed from the alignment.

### Phylogenetic analyses

We estimated species trees using the summary statistics methods STAR [21] and MP-EST [20]. The STAR method estimates the species tree from a distance matrix constructed from the average ranks of gene-coalescence events from gene trees, while MP-EST estimates the species tree from gene trees by maximizing a pseudo-likelihood function of the triplets (rooted 3-taxon statements) of the species tree. Both of these methods require user-provided gene trees for all loci and allow for some missing data. We generated maximum-likelihood gene trees for all loci (NGS and Sanger-sequenced loci) using RAxML v.7.0.4 [50] under the GTRGAMMA model. For each locus, we performed 100 bootstrap replicates, which permits error estimates to be generated on the species trees; this entire process was streamlined using a Perl-scripted pipeline developed by FTB and A. Narechania [51]. We used the Species Tree Analysis Web Server [52] to run all STAR and MP-EST analyses. The STAR and MP-EST programs were run using several different subsets of loci. For the Sanger dataset, we used the complete dataset of 5 loci and subsets of 4 loci (removing *CytB*), 4 loci (removing *COI*), and 3 (nucDNA only) loci. For the NGS dataset, we randomly subsampled the entire 377-locus dataset into 200, 100, 50, 25, 10, 5, 4, and 3-locus datasets; these subsets were run five times using different sets of random loci to determine, on average, how discordant the resulting trees were compared to the 377-locus species trees. The topological comparisons between all trees were performed using Robinson-Foulds (RF) distances in the R statistics platform [53] using the package phangorn [54]. The RF distance is a metric that determines the number of bipartitions that differ between trees to indicate the amount of topological discordance between two trees [55]. To show relative differences between the trees using the RF distances, we used the percentage of the maximum RF distance, which was calculated using 2(*n* - 2), where *n* is the number of taxa, and *n* - 2 represents the maximum number of inner branches for a rooted tree [56]. The % RF distance between trees is the ratio of the RF distance divided by the maximum RF distance. We also used the coefficient of variation for each locus-set to determine in the MP-EST and STAR analyses at what point increasing the number of loci reached diminishing returns, where additional loci no longer resulted in an improvement to the species tree; this is indicated by the lowest coefficient of variation value for each locus set. We also compared the NGS and Sanger species trees using the same numbers of loci (5, 4, and 3) to examine how similar the resulting trees are topologically when using the same numbers of loci, again using RF distances.

We next used RAxML to generate concatenated trees using the complete 377-locus NGS dataset and the complete 5-locus Sanger dataset; the Sanger dataset was partitioned by locus and codon position for the protein coding genes (the mtDNA genes were considered a single partition).. These results were compared with the explicit species-tree approaches using % RF distances. To determine how locus number affects bootstrap-support values for the species trees, a mean bootstrap value was taken across the entire tree for each of the NGS subset species trees (200, 100, 50, 25, 10, 5, 4, and 3-locus datasets) and then averaged across the five replicates for each of those subset species trees.

Although the complete 377-locus NGS dataset is too large for the full-coalescent species-tree model implemented in *BEAST [14], we ran an additional series of species trees with subsets of 15 loci to determine if this method could estimate the same topology as the full 377-locus dataset using MP-EST and STAR. A recent study of chickadee phylogenetics [7] found that among several coalescent-based species-tree inference methods, *BEAST was the most robust and consistently converged on the same tree estimated from the authors’ full dataset (40 loci) when using 15 loci. We took five random subsets of 15 NGS loci and estimated five species trees using *BEAST [18] in BEAST v1.8 [57]. In addition, we also ran *BEAST on the 5-locus Sanger dataset to determine how well it performed using a dataset similar to those typically used to infer squamate phylogenies. We used jModelTest v2.1.6 [58] with the Bayesian Information Criteria to choose the most appropriate model of sequence evolution for each locus in the *BEAST analyses, as *BEAST allows for multiple models (details available on Dryad doi:10.5061/dryad.kp400). As in our STAR and MP-EST analyses *Mimophis mahfalensis* and *Ramphiophis rubropuntatus* were used as outgroups. The *BEAST analyses were run for 2 × 10^8^ generations each using a log-normal relaxed clock model, a Yule-process speciation prior, and were sampled every 10000 generations. Tracer v1.4 [59] was used to assess stationarity for each of the runs and determine burnin. A summary table of the various methods used with the different datasets and loci is included as an additional file (Additional file 4).

To visualize congruence between resulting gene trees and the species trees, we used the program MetaTree [60]. MetaTree builds a “tree-of-trees” that shows the relationships between alternative phylogenies. This program takes user-inputted phylogenies with fixed sets of taxa to construct a tree that clusters similar topologies together, allowing the user to examine a set of trees and determine how similar topologies are to one another; here we use this program to examine whether gene trees from both the Sanger and NGS datasets are similar to the species tree topology. For our MetaTree analyses, we compared the gene trees from the Sanger dataset to the 377-locus species tree. Since *Micropisthodon* and *Rhamphiophis* were not available for all of the Sanger loci (Appendix 1), they were not included and pruned from the species tree for that comparison. For the NGS MetaTree analysis, we used subsets of 50 gene trees (enhancing visual clarity of the results) from the NGS dataset (using the 344 loci with all 25 taxa/individuals) to compare to the 377-locus species tree*.*

## Results

### Sequencing

For the Sanger dataset, sequences were aligned in Geneious® using the Geneious alignment algorithm and were checked by eye to assure no gaps in protein-coding genes. The five loci ranged from a minimum of 1.9% (*Cmos*) to a maximum of 20 % (*CytB*) uncorrected pairwise divergence across taxa (*Rag2* = 2.2 %; *Nav intron 5* = 8 %; *COI* = 17.8 %), with a mean of 10 % uncorrected pairwise divergence for all loci. For all taxa, parsimony informative sites (PI) = 26 % of sites (594/2990 total bp; from 26 PI *Cmos*–242 PI *CytB*) and for ingroup taxa only, PI = 25 % of sites (568/2990 total bp; from 20 PI *Cmos*–235 PI *CytB*). All but one locus had at least 24 of the 25 taxa (details available in additional files; Additional file 1).

For the NGS dataset, 387 loci were successfully sequenced for an average of 549,304 bp per sample before trimming. A total of 377 loci remained after filtering and orthology assessment. The resulting dataset after aligning in Mafft and trimming/masking as described above contained 513,877 alignment sites. Loci ranged in length from 424–2156 bp, with a mean locus length of 1374 bp. The matrix had very little missing data (1.5 %). All loci had at least one representative from each genus; 91.2 % of loci (344/377 loci) included all 25 terminal taxa, and 99.7 % of loci (376/377 loci) included 24 of the 25 taxa, with a single locus missing two taxa. The uncorrected pairwise divergence for loci ranged from 0.5 − 11.8 % divergent, with a mean of 3.1 % divergence across the matrix. For all taxa across all loci, PI = 8 % of sites (40147/517276 bp total; maximum = 225/1464 bp, minimum = 16/1527 bp) and for ingroup taxa, PI = 6 % of sites (30290/517276 bp total; maximum = 175/1464 bp, minimum = 12/1527 bp); the mean PI per locus = 106 bp for all taxa and 80 bp for ingroup taxa.

### Phylogenetic estimates

**Fig. 1:**
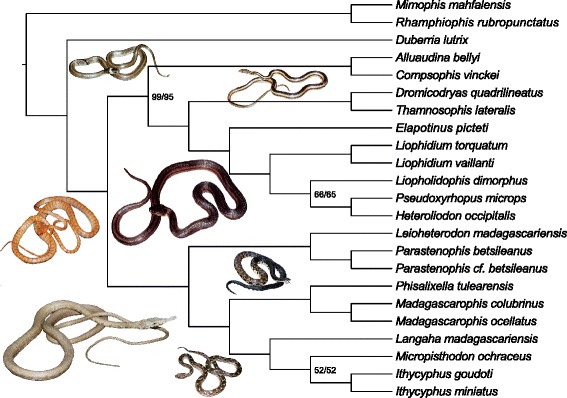
Species tree for the pseudoxyrhophiines using STAR and MP-EST (topologies identical) based on the 377-locus NGS data set. Bootstrap values are 100 for all nodes except those labeled with the STAR value first, followed by the MP-EST value. Photos CJR & SR

**Fig. 2:**
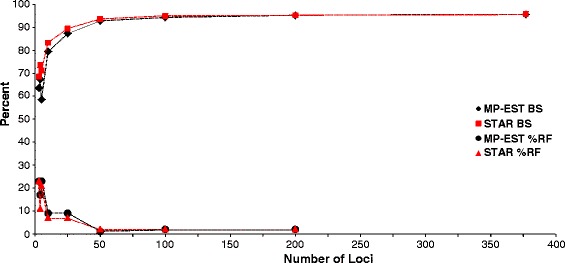
NGS dataset mean Robinson-Foulds (RF) % distances for each species tree (5 replicates with random loci subsets) with respect to the number of NGS loci used (3, 4, 5, 10, 25, 50, 100, 200) compared to the 377-locus species tree and the mean bootstrap values for each species tree (5 replicates with random loci subsets) with respect to the number of loci used (3, 4, 5, 10, 25, 50, 100, 200, 377)

The STAR and MP-EST species trees inferred for Pseudoxyrhophiinae using the complete 377-locus NGS dataset were identical (RF distance = 0 %; Fig. 1). This is our preferred tree for all subsequent topological comparisons and we consider it the species tree for the pseudoxyrhophiines throughout the remainder of the manuscript, as it was estimated using species-tree methods, included the most data and was topologically identical across a variety of methods. The concatenated 377-locus tree and all five replicates using 15 random NGS loci in *BEAST were also identical to the pseudoxyrhophiine topology of the 377-locus species tree (RF distance = 0 %), while the 5-locus Sanger *BEAST tree differed slightly from the full NGS dataset species tree (RF distance =10 %; Table 1). All *BEAST runs were found to reach stationarity by 2 × 10^8^ generations, with no trends in the trace plots suggesting that the MCMC had not converged; we discarded the first 25 % of generations as burnin. Effective sample size values were high (>200) for almost all parameters. The mean RF distances for the NGS datasets of 3–200 loci varied from 1 − 21 % (RF distances ranged from 0 − 33 % for each individual species tree estimated under each subset of loci) when compared to the 377-locus species tree. Species trees inferred using greater numbers of loci produced more similar topologies with respect to the 377-locus species tree; as locus number increased, mean RF distances generally decreased and mean bootstrap support values generally increased (Fig. 2). At ≥50 loci, the placement of a single taxon, *Liopholidophis* (Figs. 1 and 2) is responsible for all discordance among the NGS subset trees and the 377-locus species tree and the coefficients of variation calculated for MP-EST and STAR indicated that after 50 loci, increasing locus number did improve the topology with respect to matching the 377-locus species tree (all RF values and supporting data available on Dryad doi:10.5061/dryad.kp400). The species trees and concatenated trees from the Sanger datasets had RF distances similar to those from the NGS subsets of <50 loci when compared to the 377-locus species tree, with none being identical to the species tree (Table 1).

**Table 1:** Robinson-Foulds (RF) distances as % difference between trees using the Sanger (SS) and NGS datasets

Sanger Trees	SS MP-EST 5 Loci	SS STAR 5 Loci	SS *BEAST 5 Loci	377 MP-EST/STAR/Concatenated
5 Loci MP-EST	------	RF = 10 %	RF = 14 %	RF = 19 %
5 Loci STAR	RF = 10 %	------	RF = 14 %	RF = 19 %
5 Loci *BEAST	RF = 14 %	RF = 14 %	------	RF = 10 %
5 Loci Concatenated	RF = 19 %	RF = 19 %	RF = 14 %	RF = 19 %
4 Loci (no *COI*) MP-EST	RF = 10 %	RF = 5 %	RF = 19 %	RF = 24 %
4 Loci (no *COI*) STAR	RF = 10 %	RF = 10 %	RF = 24 %	RF = 33 %
4 Loci (no *CytB*) MP-EST	RF = 14 %	RF = 19 %	RF = 29 %	RF = 29 %
4 Loci (no *CytB*) STAR	RF = 10 %	RF = 0 %	RF = 14 %	RF = 19 %
3 Loci (nucDNA) MP-EST	RF = 19 %	RF = 14 %	RF = 29 %	RF = 33 %
3 Loci (nucDNA) STAR	RF = 19 %	RF = 14 %	RF = 29 %	RF = 33 %

Note that the MP-EST, STAR, and concatenated trees for the 377 loci NGS datasets were identical and so are in one column

Mean bootstrap values generally increased for the NGS species-tree analyses as the number of loci increased (Fig. 2). The bootstrap values for the 377-locus trees were high for both methods of species-tree inference, with the placement of only two taxa supported <0.95 (*Liopholidophis*, *Micropisthodon*; Fig. 1). The runs from *BEAST generally gave high posterior probability support for the same nodes, with the same two taxa (*Liopholidophis*, *Micropisthodon*) having lower posterior probability values (<0.95). Interestingly, the concatenated 377-locus tree had slightly higher support values, with only one of the nodes having a value <1.0 (*Micropisthodon + Ithycyphus*, bootstrap values = 0.39 concatenated tree/0.52 species tree). This is in contrast to the bootstrap values for the concatenated 5-locus Sanger tree, which had a lower mean bootstrap value of 0.69 across the tree versus 0.97 for the concatenated 377-locus tree.

**Fig. 3:**
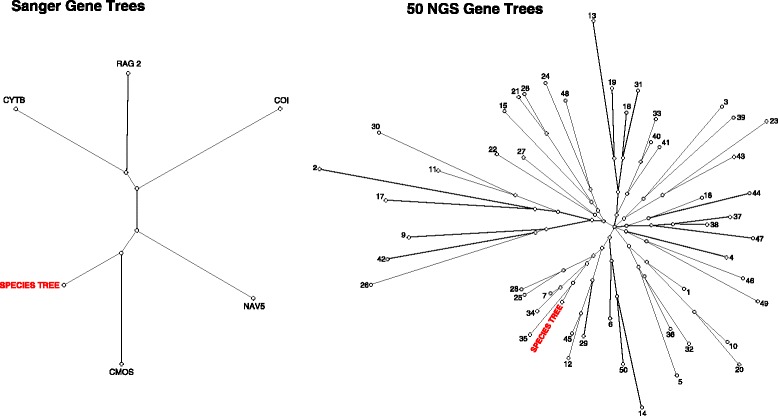
Metatree visualization showing similarity of the species tree topology (as shown in Fig. 1) with that of the 5 Sanger loci gene trees (left) and 50 NGS loci gene trees (right). The species tree is labeled for each. Sanger loci gene trees are indicated by locus name, NGS loci gene trees are indicated by number. In both data sets no gene trees were identical to the species tree

For the MetaTree comparison of the Sanger dataset to the 377-locus species tree, each Sanger gene has a unique topology and none of the Sanger gene trees were closely clustered with the 377-locus species tree, with the two mtDNA gene trees (*COI*, *CytB*) and *Rag2* gene tree being the most discordant (Fig. 3). The branches for the gene trees are also relatively long, indicating high degrees of conflict between the topologies and have few shared splits between trees [60]. For the NGS gene trees compared to the 377-locus species tree, few of the gene trees were similar to the full species tree and no gene trees were identical to the species tree; no gene tree topologies were identical to each other as well. We present these results to illustrate the large range of discordance between the species tree and the various gene trees with both the Sanger and NGS data; for brevity, one MetaTree set of 50 loci is shown in comparison with the species tree (Fig. 3; full MetaTree available on Dryad doi:10.5061/dryad.kp400).

Within pseudoxyrhophiines, the three genera with multiple species were monophyletic in all analyses. Several of the resulting clades have been recovered by other studies (e.g., *Langaha* + (*Micropisthodon* + *Ithycyphus*); [39]. Our species tree (Fig. 1) also provides well-supported placement for some enigmatic taxa such as *Elapotinus picteti* (formerly *Exallodontophis albignaci,* see [61]), which was found as the sister taxon to a clade including *Liophidium*, *Liopholidophis, Pseudoxyrhopus*, *Heteroliodon*. We also find strong support for a single monophyletic Malagasy clade of pseudoxyrhophiines with respect to the African taxon *Duberria*. We discuss the taxonomy of the group in detail below.

### Availability of supporting data

The datasets supporting the results of this article and the information pertaining to the anchored loci probes are available in the Dryad repository (doi:10.5061/dryad.kp400).

## Discussion

### Phylogenetic inference and dataset size

The number of loci required to accurately infer phylogenies has generally been addressed in a theoretical framework, but how this translates for empirical studies has not been extensively examined. Here, using datasets with varying numbers of loci and multiple phylogenetic methods, we estimate a well-supported species tree for pseudoxyrhophiine snake genera that suggests a monophyletic radiation of these snakes on Madagascar. Identical tree topologies were inferred using datasets of both 15 and ≥50 loci, but the number of loci necessary for accurate inference depends on the species-tree method.

When using the summary statistics methods STAR and MP-EST, large numbers of loci (≥50) are required to consistently produce phylogenies equal to those using all 377 loci and that the differences between topologies generally decreases as more loci are added to the analyses (Fig. 2). These results are comparable to another recent empirical study which found that increasing locus number improved topological estimates, but that ultimately the number of requisite loci plateaued [7]. There is a threshold where increasing the number of loci no longer provides greater resolution; for our dataset this is ~50 loci for both STAR and MP-EST (Fig. 2) with bootstrap-support values increasing similarly (Fig. 3). While STAR and MP-EST generate strongly supported trees for pseudoxyrhophiines, which is likely due in part to the high quality loci in our dataset, these methods may fail when gene trees are estimated from short, uninformative loci [22]. Additionally, despite the overall increase of support values as loci number increases, the position of some taxa remain poorly supported regardless of the number of loci included (*Liopholidophis*, *Micropisthodon*; Fig. 1). In particular, the instability of one taxon (*Liopholidophis*) is responsible for all topological discordance at 50 or more loci with respect to the subset NGS trees and the 377-locus tree. This suggests that some taxa may remain problematic regardless of how many loci are included. Similarly, a recent study of major snake lineages using 333 anchored phylogenomics loci and species-tree methodologies found low support values within lamprophiids, the family to which pseudoxyrhophiines belong [15]. Previous work indicates that including more individuals per species increases phylogenetic accuracy [25, 62–64] and future studies with multiple individuals per species and full species sampling per genus will determine whether more complete sampling provides better resolution for the weakly supported taxa in the pseudoxyrhophiine phylogeny.

In contrast to the summary statistics methods requiring ≥50 loci, we found that with only 15 loci, *BEAST consistently inferred the same pseudoxyrhophiine topology as the 377-locus trees from STAR and MP-EST. This suggests that for smaller datasets (e.g., <50 loci), full-coalescent methods may outperform other types of species-tree analyses requiring more loci, though the generality of this trend should be investigated. While the matching topologies of the 15-locus *BEAST analyses and all other methods using the complete dataset make us confident in the results from *BEAST here, we acknowledge that full coalescent analyses such as *BEAST should ideally include multiple individuals per terminal taxon, as this greatly improves all aspects of tree estimation [18]. Estimating population sizes in particular requires at least two individuals per species [18], as well as greater numbers of loci when compared to accurately estimating only the tree topology [7]. The results presented here may not be typical when including population-level sampling, poorly defined species, or very large numbers of taxa, since these scenarios may result in shorter branch lengths and represent recent time-scales, which likely require larger numbers of loci for good resolution and support [8]. Furthermore with a dataset that includes hundreds of species, coalescent-based methods such as *BEAST are unsuitable due to computational time and failure of parameters to converge [14, 19].

Unlike the 15-locus NGS subsets used in *BEAST, the 5-locus Sanger dataset was unable to recover the same topology as the 377-locus tree, despite these particular loci having higher average numbers of parsimony informative sites compared to the NGS loci (26 % of Sanger loci sites versus 8 % of NGS loci sites). When using STAR and MP-EST, neither the Sanger datasets nor NGS subsets of 3–5 loci resulted in species trees congruent with the 377-locus tree (Fig. 2; Table 1). Interestingly, the most congruent tree using 3–5 loci was produced using the complete 5-Sanger locus dataset in *BEAST (RF distance = 10 %; Table 1), further demonstrating that for small datasets, a full-coalescent model approach may yield the most accurate topology. Full-coalescent-model approaches may be robust to low numbers of loci for topological inference when a high proportion of gene trees match the species tree [18, 65]. However, our results indicate that datasets of ≤5 loci within any of the species-tree frameworks do not generate trees that fully correspond to the 377-locus tree for pseudoxyrhophiines (Table 1; Fig. 2). Additionally, neither of the two mtDNA gene trees (*COI*, *CytB*) closely matches the species tree, further underscoring that mtDNA gene trees, at least in this case, do not suitably represent the species tree (Fig. 3). While many studies use mtDNA gene trees as a proxy for the species tree, our results suggest that relying entirely on a single locus, whether mitochondrial or nuclear, is a potentially risky practice. It is likely that estimating credible species trees using small numbers of loci with conflicting gene-tree histories is impossible. Simulations have shown that when the majority of gene trees are highly discordant with the species tree, as many as 120 loci are necessary to recover the species tree in a coalescent-based framework [65]. Similar to our Sanger dataset results, an empirical study on lizards [66] using varying numbers of loci found that when estimating trees with four loci, *BEAST was unable to correctly infer the species tree, even when only the most informative loci were included. Therefore if a reduced set of loci are discordant it is expected that numerous additional markers are required to generate a credible species tree.

We find no topological difference for the pseudoxyrhophiines between the 377-locus concatenated tree and the 377-locus species tree (RF distance = 0 %). It is expected that when incomplete lineage sorting is low, which is likely when population sizes at the time of speciation are small and the time between speciation events is long, concatenation may accurately infer species relationships [14, 52, 67–70], especially at deep time scales [22]. However, incomplete lineage sorting can still be pervasive at deeper time scales and coalescent-based species-tree methods are able to account for this [27]. Furthermore, concatenation may overestimate branch-lengths [43] and result in positively misleading support values [65]. Finally, species-tree methods may be better suited to modeling the phylogenetic signal of very large datasets compared to concatenation (reviewed in [17]).

### Pseudoxyrhophiine generic relationships

The 377-locus species tree for the pseudoxyrhophiines presented here is well supported (Fig. 1); although several (for the most part rare) genera missing from this analysis (three Malagasy genera-*Lycodryas*, *Brygophis, Pararhadinaea*; one African genus-*Amplorhinus*; one Socotran genus-*Ditypophis*), taxonomic relationships are largely similar to those found in prior studies with respect to affinities between genera based both on morphology as well as molecular datasets, as is much of the overall structure of the tree [30, 39, 71–76]. We found two major clades of pseudoxyrhophiines: one which includes the genera *Alluaudina, Compsophis, Dromicodryas, Thamnosophis, Elapotinus*, *Liophidium, Liopholidophis, Pseudoxyrhopus*, and *Heteroliodon* and a second that includes *Leioheterodon, Parastenophis, Phisalixella, Madagascarophis, Langaha, Micropisthodon,* and *Ithycyphus*.

The major subdivision of this Malagasy snake radiation, in two approximately equally diverse clades is intriguing, in that this largely corresponds to a division of the aglyphous genera (with non-grooved teeth, nearly equal in size) and opisthoglyphous genera (with enlarged grooved teeth, at the rear of the maxillae). Historically, this tooth character was considered important for classifying snakes (e.g., [77]), and was used to organize the most recent monographic treatment of the Malagasy snakes [78]. All genera in the *Alluaudina, Compsophis, Dromicodryas, Thamnosophis, Elapotinus*, *Liophidium, Liopholidophis, Pseudoxyrhopus*, and *Heteroliodon* clade are aglyphous, with the exception of *Alluaudina* and *Compsophis.* Similarly, all genera in the *Leioheterodon, Parastenophis, Phisalixella, Madagascarophis, Langaha, Micropisthodon,* and *Ithycyphus* clade are opisthoglyphous with the exception of *Leioheterodon* and *Micropisthodon*. It is worth noting that *Leioheterodon* have enlarged rear teeth, and are known to be capable of mild envenomation [79]; and that more generally, the dentition of most of the rarer Malagasy snakes have not been recently re-evaluated. Nevertheless, our results presented here do suggest that the evolution in dentition, envenomation, and thus feeding strategies, may have influenced the early diversification of the pseudoxyrhophiine radiation, though the impact of these traits on the evolution of these groups requires further testing.

Inclusion of the continental African pseudoxyrhophiine genus *Duberria* allows us to address the biogeography of pseudoxyrhophiines in Africa and Madagascar. Many prior studies have supported a single dispersal event from continental Africa to Madagascar for the pseudoxyrhophiines, with the African taxa *Amplorhinus* and *Duberria* and the Socotran *Ditypophis* as sister taxa to the Malagasy species [38, 39, 73, 80]. This suggests the Malagasy species are monophyletic, with subsequent dispersal to the Comoros islands [81, 82]. However, not all previously used datasets/tree-methodologies, even within the same publications, support this scenario, and there is also some evidence for *Ditypophis* and/or *Duberria* nested within Malagasy taxa [30, 39, 80]. Since we do not include *Amplorhinus* or *Ditypophis* here, we cannot directly comment on their position or biogeographic origins, but our results strongly suggest that the Malagasy genera form a monophyletic group with respect to *Duberria*. While we do not show all of the summary-statistics species trees generated from the NGS subsets of loci, we note that 81/82 of those species trees place *Duberria* as the basal taxon to a monophyletic Malagasy Pseudoxyrhophiinae. Future datasets including *Amplorhinus* and *Ditypophis* will allow further exploration regarding the biogeography of both continental African and Malagasy pseudoxyrhophiines. Within the aglyphous clade, the genera *Elapotinus, Heteroliodon*, *Liopholidophis*, *Liophidium*, *Pseudoxyrhopus* have been previously referred to as the “*Pseudoxyrhopus* group” (minus *Liopholidophis* in the initial description; [71]) and were originally united based on hinged teeth and tooth replacement pattern [71]. The exact placement of *Liopholidophis* within this clade remains somewhat unclear as it is poorly supported in our species tree (bootstrap values = 66/65 %; Fig. 1), but importantly all of the discordant trees do place *Liopholidophis* within this clade. Previous molecular-based studies have indicated that *Liopholidophis* may be the sister taxon to *Liophidium* [74] but most studies [30, 39, 72, 73, 76] have found the same relationship presented here, with *Liopholidophis* being the sister taxon to *Heteroliodon* + *Pseudoxyrhopus*, and *Liophidium* in turn being the sister taxon to this inclusive clade. Although none of these prior studies have included all five genera in the same analysis, our results show the same relationships reported in previous studies [30, 39, 72–74, 76]. Additional sampling within these genera will help determine if these genera are all monophyletic or if *Heteroliodon* renders *Pseudoxyrhopus* paraphyletic, as has been previously suggested [40].

The remaining aglyphous genera include two sister taxon pairs; *Thamnophis* + *Dromicodryas*, and *Alluaudina* + *Compsophis. Thamnosophis* (formerly *Bibilava*, see [74, 83]) and *Dromicodryas* are both diurnal and active terrestrial predators, sharing external morphological similarities [78]. A sister-taxon relationship between these genera has been indicated by prior studies [30, 74, 76], and our results continue to support this relationship, as well as the recognition of *Thamnosophis* as distinct from *Liopholidophis* (see [84, 85]).

*Alluaudina* and *Compsophis* have been shown to share morphological characters with respect to hemipenes [35] and several molecular studies have found *Alluaudina* and *Compsophis* as sister taxa [39, 73] as we do here (Fig. 1). However, at least one recent molecular study [30] indicates that *Compsophis* is sister to *Ditypophis* and part of the clade that includes the mainland African/Socotran taxa (*Amplorhinus*, *Duberria*, *Ditypophis*). We find very high support for *Compsophis* being the sister taxon to *Alluaudina* (Fig. 1), rather than being placed in a clade with *Duberria*, similar to other studies where *Ditypophis, Duberria,* and/or *Amplorhinus* fall outside of the Malagasy pseudoxyrhophiines [39, 73].

Within the mostly opisthoglyphic clade of pseudoxyrhophiines, the currently monotypic *Parastenophis* was formerly a subgenus within the genus *Stenophis* (now considered paraphyletic, see [75]), which also included the currently recognized species in the genera *Phisalixella* and *Lycodryas*. However, as was suggested in a taxonomic revision of *Stenophis* [75] and noted in other molecular studies [73], it appears that *Parastenophis*, a nocturnal tree-snake, is the sister taxon to the large, diurnal and terrestrial Malagasy hognose snakes, *Leioheterodon*. The highly disparate morphology, ecology, and behavior between these sister genera [75, 79, 86], is intriguing and deserves further investigation. Although *Lycodryas* was not included in the present study, previous work [75], as well as our own unpublished results, indicate that *Lycodryas* is likely the sister taxon to *Phisalixella.* Both of these genera share very similar morphologies, and are nocturnal tree-snakes, that until recently were also included in the genus *Stenophis* [75]. As previously suggested [75] and indicated in our species tree here (Fig. 1), the sister taxon to *Phisalixella* (plus *Lycodryas*) is the similarly nocturnal and elliptical-pupiled *Madagascarophis. Madagascarophis* is a broadly distributed group of snakes in Madagascar that is also partly arboreal, but lacks the lateral body flattening and slender necks of *Phisalixella* and *Lycodryas*.

The remaining clade within the opisthoglyphic pseudoxyrhophiines contains *Langaha, Micropisthodon, Ithycyphus*, which are mostly diurnal and arboreal snakes (Fig. 1). These relationships have been supported by previous molecular studies [30, 39, 76]. Although the support for *Micropisthodon* + *Ithycyphus* is the lowest for any node in the species tree (bootstrap value = 52 %), it was consistently inferred in the species-tree analyses even when using subsets of the NGS dataset and was also found with high support values in the aforementioned studies. The behavior and ecology of *Micropisthodon* and *Langaha* are not yet well described, and the evolution and function of the unique head ornamentation in *Langaha* still not clearly understood [87, 88].

## Conclusions

The phylogeny presented here is a first step in using large-scale phylogenomic data to determine relationships for snakes of Madagascar. This phylogeny, produced using both summary statistics and full-coalescent models for species-tree estimation, is well supported and shows a monophyletic clade of Malagasy pseudoxyrhophiines. Within this radiation of snakes Madagascar, we also find strong support for two major subclades which largely correspond to the aglyphous and opisthoglyphous genera, and thus different forms of tooth venom delivery. Our results suggest that while hundreds of loci are not always essential for accurate topological inference, the method used for tree estimation may affect the resulting phylogeny and that for smaller datasets (<50 loci), full-coalescent models of tree inference are likely more accurate than summary statistics methods. Because full-coalescent model-based methods are unable to computationally handle large amounts of data, these summary statistics provide essentially the only current option for generating species trees using hundreds of loci obtained from hundreds of taxa. Importantly, our results indicate that summary statistics methods, given enough loci (~ ≥50), are able to estimate a robust species tree comparable with a full-coalescent model method. However, whether these results are applicable across different study systems is not known and it is unclear if other datasets will give similar results with respect to the numbers of loci necessary for inferring tree topologies with the methodologies used here. Using the maximum number of loci available may be the best strategy for resolving trees, though this does not account for time and expense for developing probes and sequencing these loci. Considering the alternative, where the investigator uses the minimum number of loci due to time or financial constraints, it would be difficult to know a priori which loci in combination yield trees with the most resolution and highest node support. Future NGS studies that include a far more complete sampling of species, and even populations for broadly distributed pseudoxyrhophiine taxa, will inevitably improve our ability to estimate relationships among species, and thus provide the groundwork to examine biogeography and diversification processes that generated this spectacular insular snake diversity.

## Additional files

Additional file 1:
**Samples used in analyses and which loci were successfully sequenced (NGS=Next generation sequencing; Cmos, COI, CytB, Rag2, and NAV intron 5=Sanger sequencing).** (XLSX 11 kb) Additional file 2:
**PCR and Sequencing Protocols.** All PCR reactions were 10 μL reactions consisting of 5 μL of GoTaq® Green Master Mix, 3 μL of H2O, 0.5 μL each of forward and reverse primers at a 10 μM concentration, and 1 μL of DNA extract. Samples were incubated at 96 °C for 15 min initially, 96 °C for 45 s, followed by 45 s at the appropriate temperature for the primer pair (see below), with a 72 °C extension period for 1 min. This procedure, minus the initial 15 min incubation period, was repeated for 35 cycles. Reactions were cleaned with 2 μL of ExoSap-IT® following the Exosap-IT® protocol. Sequencing reactions used the same primers as for the PCR reactions. We used the BigDye® Terminator v3.1 Cycle Sequencing Kit; each sequencing reaction consisted of 0.2 μL of ABI BigDye® Terminator Ready Reaction Mix, 1.5 μL of ABI BigDye® 5X Sequencing Buffer, 4.3 μL of H2O, 1 μL of the cleaned PCR reaction template, and 0.5 μL of the 10 μM primer for each direction. Sequencing reactions were incubated initially at 96 °C for 1 min, followed by 96 °C for 10 s, 50 °C for 5 s, and 60 °C for 1 min 15 s and repeated for 15 cycles, minus the initial 1 min incubation period; the reaction was then incubated at 96 °C for 10 s, 50 °C for 5 s, and 60 °C for 1 min 30 s and repeated for 6 cycles; the reaction was then incubated at 96 °C for 10 s, 50 °C for 5 s, and 60 °C for 2 min and repeated for 5 cycles. Sequencing reactions were cleaned prior to sequencing using ethanol precipitation. (PDF 155 kb) Additional file 3:
**Summary of the Anchored Phylogenomics assembly results.** (XLSX 17 kb) Additional file 4:
**Tree estimation methods, with the dataset type (Sanger and NGS) and the number(s) of loci used with each method.** (PDF 4 kb)
