# Supplementary material for: Comparing species tree estimation with large anchored phylogenomic and small Sanger-sequenced molecular datasets: an empirical study on Malagasy pseudoxyrhophiine snakes
Source: BMC Evol Biol. 2015 Oct 12;15:221. doi: 10.1186/s12862-015-0503-1 (PMC4603904; doi:10.1186/s12862-015-0503-1)
Supplement: Additional file 2: — PCR and Sequencing Protocols. All PCR reactions were 10 μL reactions consisting of 5 μL of GoTaq® Green Master Mix, 3 μL of H2O, 0.5 μL each of forward and reverse primers at a 10 μM concentration, and 1 μL of DNA extract. Samples were incubated at 96 °C for 15 min initially, 96 °C for 45 s, followed by 45 s at the appropriate temperature for the primer pair (see below), with a 72 °C extension period for 1 min. This procedure, minus the initial 15 min incubation period, was repeated for 35 cycles. Reactions were cleaned with 2 μL of ExoSap-IT® following the Exosap-IT® protocol. Sequencing reactions used the same primers as for the PCR reactions. We used the BigDye® Terminator v3.1 Cycle Sequencing Kit; each sequencing reaction consisted of 0.2 μL of ABI BigDye® Terminator Ready Reaction Mix, 1.5 μL of ABI BigDye® 5X Sequencing Buffer, 4.3 μL of H2O, 1 μL of the cleaned PCR reaction template, and 0.5 μL of the 10 μM primer for each direction. Sequencing reactions were incubated initially at 96 °C for 1 min, followed by 96 °C for 10 s, 50 °C for 5 s, and 60 °C for 1 min 15 s and repeated for 15 cycles, minus the initial 1 min incubation period; the reaction was then incubated at 96 °C for 10 s, 50 °C for 5 s, and 60 °C for 1 min 30 s and repeated for 6 cycles; the reaction was then incubated at 96 °C for 10 s, 50 °C for 5 s, and 60 °C for 2 min and repeated for 5 cycles. Sequencing reactions were cleaned prior to sequencing using ethanol precipitation. (PDF 155 kb) [file 12862_2015_503_MOESM2_ESM.pdf]

Additional file 2. PCR and Sequencing Protocols. All PCR reactions were 10µL reactions consisting of 5µL of GoTaq® Green Master Mix, 3µL of H_2O, 0.5µL each of forward and reverse primers at a 10 µM concentration, and 1µL of DNA extract. Samples were incubated at 96° C for 15 min initially, 96° C for 45 s, followed by 45 s at the appropriate temperature for the primer pair (see below), with a 72° C extension period for 1 minute. This procedure, minus the initial 15 minute incubation period, was repeated for 35 cycles. Reactions were cleaned with 2µL of ExoSap-IT® following the Exosap-IT® protocol. Sequencing reactions used the same primers as for the PCR reactions. We used the BigDye® Terminator v3.1 Cycle Sequencing Kit; each sequencing reaction consisted of 0.2µL of ABI BigDye® Terminator Ready Reaction Mix, 1.5 µL of ABI BigDye® 5X Sequencing Buffer, 4.3µL of H_2O, 1 µL of the cleaned PCR reaction template, and 0.5µL of the 10µM primer for each direction. Sequencing reactions were incubated initially at 96° C for 1 minute, followed by 96° C for 10 s, 50° C for 5 s, and 60° C for 1 min 15 s and repeated for 15 cycles, minus the initial 1 minute incubation period; the reaction was then incubated at 96° C for 10 s, 50° C for 5 s, and 60° C for 1 min 30 s and repeated for 6 cycles; the reaction was then incubated at 96° C for 10 s, 50° C for 5 s, and 60° C for 2 min and repeated for 5 cycles. Sequencing reactions were cleaned prior to sequencing using ethanol precipitation.

**Locus**	**PCR Temp.**	**Primer**		**Reference**
CMOS	48.0^o C	S77	5’- CAT GGA CTG GGA TCA GTT ATG-3’	Lawson et al. 2005
		S78	5’- CCT TGG GTG TGA TTT TCT CAC CT-3’	Lawson et al. 2005
COI	48.0^o C	RepCOI-F	5’- TNT TMT CAA CNA ACC ACA AAG A-3’	Nagy et al. 2012
		RepCOI-R	5’- ACT TCT GGR TGK CCA AAR AAT CA-3’	Nagy et al. 2012
CYTB	45.5^o C	L14910	5’- GAC CTG TGA TMT GAA AAA CCA YCG TTG T-3’	Burbrink et al. 2000
		LycodryasG3R	5’- TGG AAT GGR ATT TTR TCG AT-3’	Developed for this project
RAG2	48.0^o C	L562	5’- CCT RAD GCC AGA TAT GGY CAT AC-3’	Vidal and Hedges 2005
		H1306	5’- GHG AAY TCC TCT GAR TCT TC-3’	Vidal and Hedges 2005
NAV intron 5	48.0^o C	NAV5F	5’- GGG CAA CGT CTC TGC TCT AC-3’	Geffeney et al. 2005
		NAV6R	5’-GCA AGT TCC CCA TGA ACA GT-3’	Geffeney et al. 2005
