# Supplementary material for: Comparing species tree estimation with large anchored phylogenomic and small Sanger-sequenced molecular datasets: an empirical study on Malagasy pseudoxyrhophiine snakes
Source: BMC Evol Biol. 2015 Oct 12;15:221. doi: 10.1186/s12862-015-0503-1 (PMC4603904; doi:10.1186/s12862-015-0503-1)
Supplement: Additional file 4: — Tree estimation methods, with the dataset type (Sanger and NGS) and the number(s) of loci used with each method. (PDF 4 kb) [file 12862_2015_503_MOESM4_ESM.pdf]

Additional file 4. Tree estimation methods, with the dataset type (Sanger and NGS) and the number(s) of loci used with each method.

Tree Estimation Method	Sanger dataset	NGS dataset
MP-EST	3 (nucDNA), 4 (no CytB), 4 (no COI), 5	3, 4, 5, 10, 25, 50, 100, 200, 377
STAR	3 (nucDNA), 4 (no CytB), 4 (no COI), 5	3, 4, 5, 10, 25, 50, 100, 200, 377
*BEAST	5	15
RaxML Concatentation	5	377
